# Esophageal cancer responsive to the combination of immune cell therapy and low-dose nivolumab: two case reports

**DOI:** 10.1186/s13256-020-02634-z

**Published:** 2021-04-08

**Authors:** Rishu Takimoto, Takashi Kamigaki, Takuji Gotoda, Toshimi Takahashi, Sachiko Okada, Hiroshi Ibe, Eri Oguma, Shigenori Goto

**Affiliations:** 1 Seta Clinic Group, New Surugadai Bldg. 3F, 2-1-45 Kandasurugadai, Chiyoda-ku, Tokyo, 101-0062 Japan; 2 Department of Next-Generation Cell and Immune Therapy, Graduate School of Medicine, Juntendo University, Yamanoue Bldg. 7F, 3-2-6 Hongo, Bunkyo-ku, Tokyo, 113-8421 Japan; 3 Division of Gastroenterology and Hepatology, Nihon University, 1-6, Kandasurugadai, Chiyoda-ku, Tokyo, 101-8309 Japan

**Keywords:** Immune cell therapy, Esophageal cancer, Immune checkpoint inhibitor, αβT cell therapy, Dendritic cell vaccine

## Abstract

**Background:**

Blocking the programmed death 1 pathway by immune checkpoint inhibitors induces dramatic antitumor activity in patients with malignant tumors. However, the clinical response to immune checkpoint inhibitors remains limited owing to the patients’ immunological status, such as the number of lymphocytes, programmed death ligand 1 expression, and tumor mutation burden. In this study, we successfully treated two patients with advanced esophageal cancer who responded to the combination of adoptive immune cell therapy and a low-dose immune checkpoint inhibitor, nivolumab.

**Case presentation:**

Two Asian (Japanese) patients with advanced esophageal cancer who were resistant to conventional chemoradiation therapy were referred to our hospital for immune therapy. Case 1 was a 66-year-old woman who was diagnosed as having esophageal cancer. She received concurrent chemoradiation therapy and then underwent subtotal esophagectomy, after which she became cancer free. However, she relapsed, and cancer cells were found in the lung and lymph nodes 6 months later. She enrolled in a clinical trial at our institution (clinical trial number UMIN000028756). She received adoptive immune cell therapy twice at a 2-week interval followed by low-dose nivolumab with adoptive immune cell therapy four times at 2-week intervals. A follow-up computed tomography scan showed partial response, with mass reduction of the metastatic lung and mediastinal lesions. Case 2 was a 77-year-old man. He received concurrent chemoradiation therapy with fluoropyrimidine/platinum, and gastroscopy revealed complete remission of esophageal cancer. He was disease free for 5 months, but routine computed tomography revealed multiple metastases in his lungs and lymph nodes. He visited our clinic to receive adoptive immune cell therapy and immune checkpoint inhibitor combination therapy. Radiographic evidence showed continuous improvement of lesions. There was no evidence of severe adverse events during the combination therapy.

**Conclusion:**

The combination of adoptive immune cell therapy and an immune checkpoint inhibitor might be a possible treatment strategy for advanced esophageal cancer.

*Trial registration* UMIN000028756. Registered 14 September 2017

## Background

Esophageal cancer is an aggressive malignancy and the most common cause of cancer-related death worldwide [1]. Although treatment strategies such as surgery, chemotherapy, radiotherapy, and chemoradiotherapy have been developed in recent years, the prognosis for patients with recurrent or advanced-stage esophageal cancer remains poor [2]. The limited improvement in treatment outcome obtained by conventional therapies has prompted the search for innovative strategies for the treatment of this cancer, especially molecular or immune-targeting treatments.

The programmed death 1 (PD-1) pathway serves as a checkpoint to limit T-cell-mediated immune responses. Two ligands, programmed death ligand 1 (PD-L1) and programmed death ligand 2 (PDL-L2), engage the PD-1 receptor and induce PD-1 signaling and associated T-cell exhaustion, resulting in reversible inhibition of T-cell activation and proliferation [3]. Tumor cells can co-opt the PD-1 pathway to evade immune responses by expressing PD-L1 on the cell surface and engaging PD-1 receptor-positive immune effector cells [4]. Thus, PD-1 and PD-L1 have attracted considerable attention for their roles in tumor immunology and as immune-based therapeutic targets [3, 5]. A number of clinical trials of PD-1/PD-L1 signal-blockade agents as immune checkpoint inhibitors (ICIs) have recently demonstrated dramatic antitumor efficacy in patients with numerous types of malignancy, including esophageal cancer [6, 7].

Although PD-L1 expression in tumor tissues prior to treatment correlates with clinical outcomes, the density of tumor-infiltrating lymphocytes (TILs) in the invaded margin of the tumor may better predict the response to anti-PD-1/PD-L1 therapy [8]. Recent research has shown four different types of tumor microenvironment based on the presence or absence of TILs and PD-L1 expression [9]. Tumors that are both PD-L1 and TIL positive are most likely to benefit from single-agent anti-PD-1/PD-L1 blockade because such tumors possess preexisting TILs that are turned off by PD-L1 engagement. Thus, better understanding of PD-L1 expression and TIL status in esophageal cancer tissues may have considerable clinical implications.

Adoptive T-cell therapy using TILs has been found to mediate durable, complete cancer regression in patients with melanoma and epithelial cancers [10–12]. Collectively, these responses were likely based on the recognition of unique, patient-specific mutated neoantigens through the T-cell receptor (TCR) [12]. However, isolation of TILs from cancer tissue is not always feasible. It was recently demonstrated that peripheral blood lymphocytes contained TIL-like cells recognizing tumor-specific antigens and could be a source of noninvasive options for immune cell therapy [13]. Previously, we and others reported that adoptive T-cell therapy using peripheral blood mononuclear cells (adoptive cell therapy, ACT), which are activated and proliferated through a culture process involving stimulation with an immobilized anti-CD-3 antibody and interleukin-2 (IL-2), has shown certain efficacy against various cancers without severe adverse events [14, 15], indicating that the efficacy of ACT might partially be derived from TILs associated with peripheral blood cells.

Although ICIs have great potential for cancer immunotherapy, their efficacy is still limited by some toxicities. Synergies between ICI therapy and other immunotherapies including cancer vaccines or ACT are currently being investigated in several clinical trials [15]. Here, we report the cases of two patients with advanced esophageal cancer who were successfully treated and responded to the combination of ACT and an ICI, nivolumab, suggesting that this combination might be a possible treatment strategy for advanced esophageal cancer.

## Case presentation

### Case 1

**Fig. 1:**
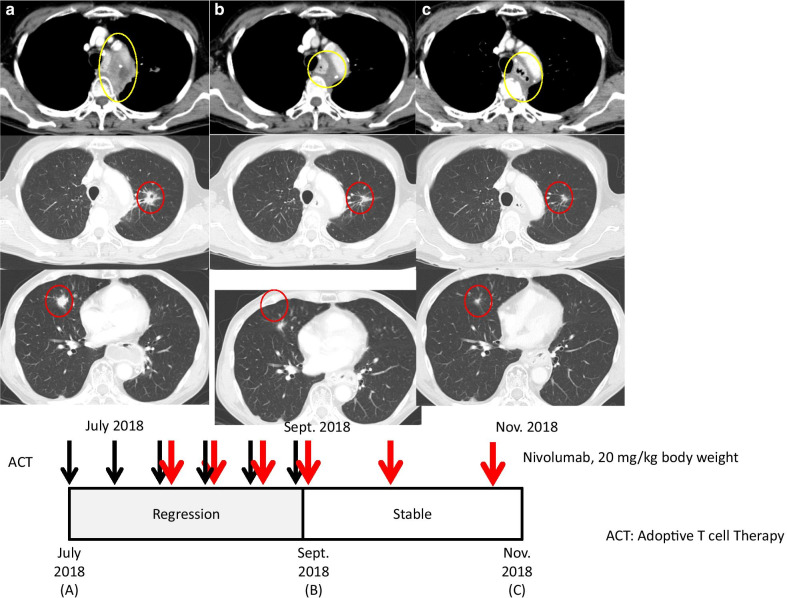
Clinical course of case 1. Axial computed tomography images corresponding to the timelines of therapy and disease status. Yellow circles indicate the mediastinal lesion, and red circles indicate lung metastatic lesions. **a** (top) status before treatment with nivolumab. **b** Regression of both mediastinal and lung metastatic lesions after combination adoptive cell therapy (ACT) and immune checkpoint inhibitor (ICI) therapy. Responses were durable during maintenance therapy with the ICI (**c**). Black and red arrows indicate ACT and nivolumab administration, respectively (bottom)

A female Asian (Japanese) patient was diagnosed as having esophageal cancer in February 2016 at 66 years of age. Biopsy and computed tomography (CT) revealed squamous cell carcinoma of the esophagus at clinical stage IV (UICC, T4N2M0). She received concurrent chemoradiation therapy (CCRT) with fluoropyrimidine/platinum from March to April 2016, then underwent subtotal esophagectomy in June 2016. Histopathological analysis of surgical tumor specimens revealed that her esophageal cancer was at clinical stage II (UICC, T3N0M0). She remained disease free until November 2016, when routine CT revealed a new pulmonary nodule and mediastinal lymph node swelling, 2.0 cm in diameter, in her left lung. She visited our clinic to receive ACT in December 2016 after radiotherapy for the lymph node metastasis in her lung from January to February 2017. During radiotherapy, she also received ACT using αβT lymphocytes four times at 2–3-week intervals until April 2017. Then chemotherapy using fluoropyrimidine/platinum/docetaxel was administered until January 2018. However, routine CT revealed multiple lung metastases in February 2018. In July 2018, she enrolled in a clinical trial at our institution (clinical trial number UMIN000028756) (Fig. 1a). Before starting the clinical trial, we evaluated PD-L1 expression level using her tumor specimens and found that the PD-L1 expression level in the tumor tissue was less than 1%. The tumor was negative for microsatellite instability (MSI; data not shown). She received ACT twice at a 2-week interval, followed by nivolumab at a dose of 0.3 mg/kg body weight with ACT four times at 2-week intervals, as part of induction therapy. A follow-up CT scan on 19 September 2018 (9 weeks after nivolumab initiation) showed partial response, with 48% mass reduction of the lung metastases and mediastinal lesion (Fig. 1b). She was allowed to continue with nivolumab treatment at a dose of 40 mg/kg body weight as maintenance therapy at 3-week intervals. Over her clinical course in 2018, there was radiographic evidence of slight improvement (Fig. 1c), and maintenance nivolumab therapy was continued because the patient was clinically well and alive. Mild, asymptomatic hypothyroidism developed, which required thyroid hormone supplementation, but she showed no other clinically significant treatment-related toxicity.

### Case 2

**Fig. 2:**
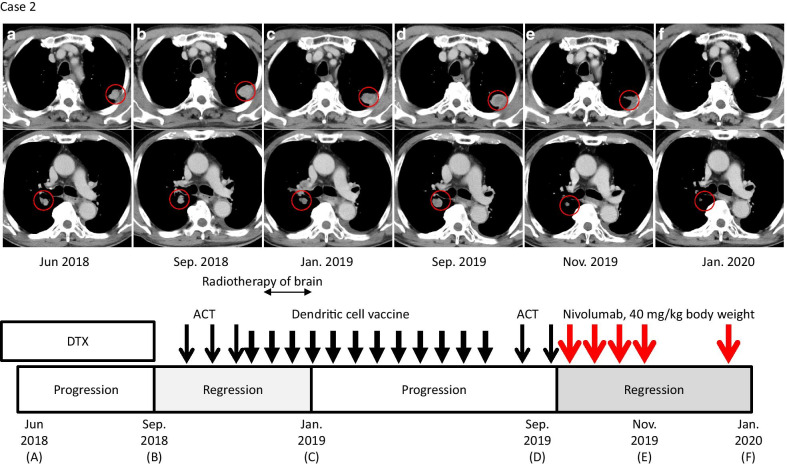
Clinical course of case 2. Axial CT images corresponding to the timelines of therapy and disease status. Red circles indicate the lung and hilar metastatic lesions. **a** (top) Status during administration of docetaxel (DTX). **b** Progression of lung metastasis before treatment with adoptive cell therapy (ACT). **c** Regression of lung metastasis after treatment with ACT and dendritic cell (DC) vaccine. **d** Progression of lung and hilar lymph node metastases before administration of nivolumab. **e** Regression of lung metastasis 8 weeks after immune checkpoint inhibitor (ICI) and ACT combination treatment. Responses were durable during maintenance therapy with the ICI (**f**). Narrow black arrows, bold black arrows, and red arrows indicate ACT, DC vaccine, and nivolumab administration, respectively (bottom)

A male Asian (Japanese) patient was diagnosed as having esophageal cancer in June 2016 at 77 years of age. CT and biopsy of specimens revealed squamous carcinoma of the esophagus at clinical stage IV (UICC, T4N2M1). He received CCRT with fluoropyrimidine/platinum from July to November 2017. In December 2017, gastroscopy revealed complete remission of esophageal cancer. He remained disease free until April 2018, when routine CT revealed multiple metastases in his bilateral lungs and lymph nodes of the right hilum (Fig. 2a). He was administered docetaxel as second-line chemotherapy from May to September 2018, but his lung metastases were found to have progressed (Fig. 2b). He visited our clinic to receive ACT in October 2018, followed by ACT using αβT lymphocytes three times at 2–3-week intervals until December 2018. He then received dendritic cells (DCs) pulsed with MUC1, MAGE3, and survivin, which were expressed on his tumor cells as tumor antigens, 12 times at 2–3-week intervals from 26 December 2018 to 28 June 2019 (Fig. 2). During ACT and pulsed DC therapy, he developed brain metastasis in November 2018 and underwent stereotactic radiosurgery for brain metastasis. Routine CT revealed that the sizes of multiple lung and lymph node metastatic lesions were reduced, which were evaluated as partial response on the basis of Response Evaluation Criteria in Solid Tumors (RECIST) version 1.1 in January 2019 (Fig. 2c). Furthermore, the sizes of brain metastatic lesions were decreased in April 2019. In September 2019, follow-up CT revealed regrowth of metastatic lesions in the lung and hilar lymph nodes, and he started to receive ACT once a month (Fig. 2d). Immunohistochemical staining revealed that the PD-L1 expression level in the tumor was 1%, and the tumor was negative for MSI (data not shown). Following ACT, he received nivolumab at a dose of 0.6 mg/kg body weight four times at 2-week intervals as part of induction therapy. A follow-up CT scan on 27 November 2019 (8 weeks after nivolumab initiation) showed partial response, with 60% reduction of the lung mass and hilar lymph node swelling (Fig. 2e). He was allowed to continue with nivolumab treatment at a dose of 40 mg/kg body weight as maintenance therapy at 3-week intervals. Over his clinical course in 2019, radiographic evidence showed continuous improvement of lesions, and his treatment was continued with maintenance nivolumab therapy (Fig. 2f) because the patient was clinically well and alive. There was no evidence of adverse events during combination therapy.

### Flow cytometric analysis of patients’ peripheral blood mononuclear cells before and after combination immunotherapy with ACT and ICI

**Fig. 3:**
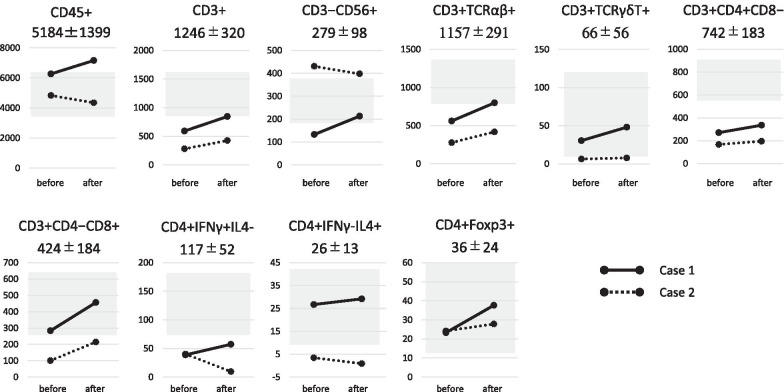
Flow cytometry of peripheral blood mononuclear cells (PBMCs) before and 2 weeks after combination therapy with ICI and ACT. The phenotype of PBMCs was analyzed as described in “Patients and methods.” The phenotype and mean ± standard deviation (shaded box) in healthy subjects are shown in each graph. The solid line indicates the number of cells in case 1, and the dotted line indicates that in case 2

The total number of cells used for ACT ranged from 7.3 to 10.9 × 10^9^ (average 8.3 ± 1.7 × 10^9^ cells/infusion) in case 1 and from 3.9 to 7.8 × 10^9^ (average 6.1 ± 1.7 × 10^9^ cells/infusion) in case 2, and the characteristics of αβT cells prepared from each patient were not significantly changed at the first and fourth cultivation (data not shown). The numbers of white blood cells (WBCs) and CD45^+^ leukocytes in peripheral blood did not change after ICI and ACT combination immunotherapy (Fig. 3). The numbers of CD3^+^, TCRαβ^+^, TCRγδT^+^, CD4^+^CD8^−^T, and CD4^−^CD8^+^T cells were significantly lower than those in healthy subjects before ACT [16], and increased after ICI and ACT combination immunotherapy (Fig. 3). There were no significant differences in the numbers of CD3^−^CD56^+^, IFN-γ+IL4^−^ (Th1), IFN-γ-IL4^+^ (Th2), and Foxp3+ (Treg) cells among the CD3^+^CD4^+^ subset cells (Fig. 3).

## Discussion and conclusion

We describe herein two cases of patients treated with the combination of ACT and an ICI; one patient was treated concurrently with ACT and an ICI, and the other patient obtained a partial response by administration of ACT and a DC vaccine followed by ICI administration for recurrence.

Although grade 1 hypothyroidism occurred in case 1 that required thyroid hormone supplementation, we observed no further severe adverse events in either case. Since we assumed that combination therapy with ACT and the ICI might increase the incidence and severity of adverse events, we used a much lower dose of nivolumab than the standard dose, that is, 0.3 or 0.6 mg/kg body weight (20 mg/kg body weight or 40 mg/kg body weight), respectively. The standard dose of nivolumab used in cancer therapy is usually 240 mg/kg body weight, so the dose administered in this trial was one-sixth or one-twelfth of the standard dose of nivolumab, for which no sufficient clinical data have been reported for evaluating the efficacy of nivolumab for esophageal cancer. However, it has been shown by *in vitro* analysis that even a low dose of nivolumab, 0.3 mg/kg body weight, was sufficient to inhibit PD-L1/PD-1 association [17].

Several biomarkers that can predict the clinical response of nivolumab have been reported. PD-L1 expression is one of the candidates, since a number of gastrointestinal cancers overexpress this molecule [18, 19]. Although PD-L1 expression determined by immunohistochemical staining has been correlated with prognosis and response to ICIs in several studies [17, 18], other studies demonstrated ICI efficacy in patients deemed to be PD-L1 negative [20]. Thus, the true relationship between PD-L1 expression and clinical efficacy has not yet been elucidated. Tumor mutation burden (TMB) has been demonstrated to be significantly associated with PD-1 and the PD-L1 blocking response. Cancers that have a higher TMB, that is, a higher neoantigen exposure to the immune system, seem more likely to respond to ICIs [21]. In both patients reported herein, analysis of their tumor specimens showed microsatellite stability and a PDL-1 expression level of 1% or lower. TILs are also found to be an independent marker for prolonging progression-free survival and overall survival in esophageal cancer, thus indicating the critical role of T cells in tumor immunity [22]. Nevertheless, these markers do not always determine the treatment response to ICIs, suggesting that other factors, such as host immunity, might affect the clinical response to ICIs. For example, an association has been demonstrated between pretreatment lymphocyte count and response to ICIs: patients with higher baseline lymphocyte counts showed better clinical benefits from ICIs [23]. Lymphocytes in peripheral blood have been reported to include T cells targeting neoantigens derived from tumor cells [13, 24]. Thus, an adequate immune status of T cells in patients is necessary to obtain better efficacy of ICIs. Our previous studies revealed that the T cell immune status was impaired in advanced cancer patients and it was restored by ACT, suggesting the beneficial effect of combination therapy with ICIs and ACT [16, 24]. Compatible with these observations, flow cytometric analysis revealed that the numbers of CD3^+^T lymphocytes and their subsets, including TCRαβ^+^, TCRγδT^+^, CD4^+^CD8^−^T, and CD4^−^CD8^+^T cells, increased after ICI and ACT combination therapy in both patients (Fig. 3), and it might lead to favorable responses to ICIs.

Although the dose of nivolumab given to both patients was very low, it remains unclear whether clinical responses could be obtained at a much lower dose of the ICI alone or the combination of ACT and the ICI. A controlled study is necessary with a large number of patients to clarify this issue. In the case that even a very low dose of nivolumab is found to be effective, dose escalation studies may be required to reevaluate the doses of ICIs for cancer treatment.

## Patients and methods

### Patients

This study was conducted from September 2017 to March 2019. The patients were administered ACT twice at a 2–3-week interval followed by 0.3–0.6 mg/kg nivolumab and ACT four times at 2-week intervals. The institutional review board of the hospital approved the study, and written informed consent was obtained from the patients (clinical study number, UMIN000028756: an exploratory clinical trial on the safety of combination therapy with effector cell therapy and immune checkpoint inhibitors for patients with malignant tumor).

### Preparation for ACT

Activated lymphocytes were generated as previously described [14]. In brief, peripheral blood mononuclear cells (PBMCs) were isolated from the patients’ peripheral blood using a Vacutainer (Becton, Dickinson and Company, Franklin Lakes, NJ, USA). The PBMCs were activated in a culture flask with an immobilized monoclonal antibody to CD3 (Jansen-Kyowa, Tokyo, Japan) in HyMedium 930 (Kohjin Bio, Saitama, Japan) containing 1% autologous serum. The PBMCs were then cultured for 14 days with 700 IU/ml IL-2 (Proleukin^®^; Chiron, Amsterdam, Netherlands), after which 3–10 × 10^9^ cells were harvested and suspended in 50 ml normal saline for intravenous injection. To prepare a dendritic cell (DC) vaccine, PBMCs were collected from the patients by leukapheresis and allowed to adhere to a plastic culture flask. The adherent cell fraction was used for DC culture for 6 days in a medium supplemented with 50 ng/ml IL-4 (Primmune Corp., Osaka, Japan) and 50 ng/ml granulocyte macrophage colony-stimulating factor (GM-CSF) (Primmune Corp.) to generate immature DCs. The DCs were pulsed with antigenic tumor-specific peptides or an autologous tumor lysate and allowed to mature for 24 h. After the culture, 1–10 × 10^6^ mature DCs were harvested and suspended in 1 ml normal saline for subcutaneous injection, then cryopreserved until the day of administration.

### Flow cytometry of PBMCs

Heparinized whole blood was collected from the patients. The phenotype of PBMCs was analyzed by whole-blood staining with OptiLyse C lysis solution [16]. Absolute cell number was determined using Flow-Count™ fluorospheres as internal standard beads. OptiLyse C, Flow-Count beads, and monoclonal antibodies (mAbs) against CD3, CD4, CD8, CD14, CD16, CD19, CD45, CD56, TCR pan αβ, TCR pan γδ, and TCR Vγ9 were purchased from Beckman Coulter (Brea, CA, USA). Lymphoprep™ (Axis-Shield PoC AS, Oslo, Norway) was used with gradient centrifugation to isolate the PBMCs. For Foxp3 staining, the PBMCs were fixed and permeabilized using a fixation/permeabilization kit (BioLegend, San Diego, CA, USA) in accordance with the manufacturer's protocol, and Foxp3 was stained with anti-Foxp3 mAb (clone 259D, BioLegend). For intracellular cytokine production assay, the PBMCs were suspended in a conditioned medium supplemented with 10% heat-inactivated fetal bovine serum (Invitrogen, Grand Island, NY, USA) containing phorbol 12-myristate 13-acetate (Sigma-Aldrich, St. Louis, MO, USA), ionomycin (Sigma-Aldrich), and brefeldin A (Sigma-Aldrich). The cells were incubated at 37 °C in a humidified atmosphere with 5% CO_2_ for 4 h for the IFN-γ/IL-4 assay. After the activated cells were fixed and permeabilized, intracellular cytokines were stained with an anti-IFN- γ or anti-IL-4 (Beckman Coulter) antibody. A Cytomics FC500 or a Gallios flow cytometer (Beckman Coulter) was used for data acquisition, and the data were analyzed using CXP or Kaluza software (Beckman Coulter).

### Immunohistochemistry

To measure the PD-L1 expression level in cancer tissue, PD-L1 IHC 22C3 pharmDx (Agilent Technologies, Santa Clara, CA, USA) was used in accordance with the manufacturer’s instructions.

### Microsatellite instability

Microsatellite instability (MSI) was determined using a modified version of the pentaplex polymerase chain reaction (PCR) assay as described by Buhard *et al*. [25] using five markers (NR-21, BAT-26, BAT-25, NR-24, and MONO-27) and analyzed using an ABI PRISM 3100 genetic analyzer (Applied Biosystems, Foster City, CA, USA) in accordance with the manufacturer’s instructions.

## Data Availability

The datasets used and/or analyzed during the current study are available from the corresponding author on reasonable request.

## Abbreviations

PD-1: Programmed death 1
ICI: Immune checkpoint inhibitor
ACT: Adoptive immune cell therapy
PD-L1 and PD-L2: Programmed death ligand-1 and programmed death ligand-2, respectively
TIL: Tumor-infiltrating lymphocyte
TCL: T-cell receptor
IL-2: Interleukin-2
PBMC: Peripheral blood mononuclear cell
DC: Dendritic cell
GM-CSF: Granulocyte macrophage colony-stimulating factor
MSI: Microsatellite instability
CT: Computed tomography
CCRT: Concurrent chemoradiation therapy
DTX: Docetaxel
